# Walking Dynamics, User Variability, and Window Size Effects in FGO-Based Smartphone PDR+GNSS Fusion

**DOI:** 10.3390/s26020431

**Published:** 2026-01-09

**Authors:** Amjad Hussain Magsi, Luis Enrique Díez

**Affiliations:** Faculty of Engineering, University of Deusto, Avda. Universidades 24, 48007 Bilbao, Spain; luis.enrique.diez@deusto.es

**Keywords:** factor graph optimization, fusion, GNSS, Kalman filter, PDR, sliding window, smartphone

## Abstract

The performance of smartphone-based pedestrian positioning strongly depends on the GNSS signal quality, the motion dynamics that influence PDR accuracy, and the way both sources of information are fused. While recent studies have shown the benefits of Factor Graph Optimization (FGO) for Pedestrian Dead Reckoning (PDR) Global Navigation Satellite Systems (GNSS) fusion, the interaction between human motion, PDR errors, and FGO window configuration has not been systematically examined. This work investigates how walking dynamics affect the optimal configuration of sliding-window FGO, and to what extent FGO mitigates motion-dependent PDR errors compared with the Kalman Filter (KF). Using data collected from ten pedestrians performing four motion types (slow walking, normal walking, jogging, and running), we analyze: (1) the relationship between walking speed and the FGO window size required to achieve stable positioning accuracy, and (2) the ability of FGO to suppress PDR outliers arising from motion irregularities across different users. The results show that a window size of around 10 poses offers the best overall balance between accuracy and computational load, providing substantial improvement over SWFGO with a 1-pose window and approaching the accuracy of batch FGO at a fraction of its cost. Increasing the window further to 30 poses yields only marginal accuracy gains while increasing computation, and this trend is consistent across all motion types. Additionally, FGO and SWFGO reduce PDR-induced outliers more effectively than KF across all users and motions, demonstrating improved robustness under gait variability and transient disturbances.

## 1. Introduction

Accurate pedestrian positioning remains challenging due to the inherent limitations of GNSS. In general, GNSS suffers from multipath, signal blockage, atmospheric delays, and non-line-of-sight (NLOS) effects, which introduce significant fluctuations in positioning accuracy. These issues become even more severe on smartphones, where low-cost GNSS chipsets, reduced antenna quality, and limited signal-processing capabilities further degrade measurement reliability, often producing intermittent jumps, biased pseudoranges, and unstable fixes. As a result, GNSS alone is insufficient to provide reliable and continuous pedestrian localization, especially in dense urban environments [1].

To overcome this, GNSS is commonly complemented with inertial sensing. However, a pure Inertial Navigation System (INS) is not suitable for smartphone-based pedestrian tracking. Although high-quality INS can maintain accurate positioning over short distances, the low-cost MEMS IMUs in smartphones exhibit large biases and noise that cause rapid drift. Moreover, pedestrian INS algorithms typically rely on Zero-Velocity Updates (ZUPT) to constrain drift during stance phases, but ZUPT cannot be applied to smartphones because the device is not foot-mounted and does not experience a consistent stationary interval at each step. Without ZUPT, standalone inertial integration diverges within seconds [2].

For these reasons, pedestrian navigation systems widely adopt Pedestrian Dead Reckoning (PDR), which extracts relative motion from step detection, step length estimation, and heading estimation instead of performing full inertial integration. PDR provides stable short-term motion increments but still accumulates drift in heading and step length, particularly during turns, speed changes, or irregular gait patterns. Consequently, robust pedestrian navigation requires the fusion of GNSS and PDR because GNSS constrains long-term drift, while PDR maintains local continuity and provides motion information during GNSS-degraded conditions [3]. Given the importance of PDR+GNSS fusion, recent research has extensively explored different integration strategies to improve robustness and accuracy under diverse motion conditions and smartphone constraints.

A large body of work [3,4,5,6,7], including our previous studies on FGO vs. KF fusion for different pedestrian motions [8], FGO performance under PDR errors [9], and windowing strategies for smartphone-based PDR+GNSS fusion [10], has investigated specific components of the PDR+GNSS architecture. These studies established that FGO can outperform KF in both walking and running scenarios, that PDR errors vary substantially across users, and that sliding-window strategies can reduce computational burden without severely compromising accuracy. However, one important aspect has not yet been addressed: Most existing studies on FGO-based PDR+GNSS fusion focus on algorithmic comparisons or average positioning accuracy using fixed window configurations, often evaluated under limited motion conditions. In particular, prior work typically considers walking or running scenarios without explicitly analyzing how pedestrian motion dynamics and inter-user gait variability influence PDR uncertainty and the optimal choice of sliding-window size. As a result, the interaction between walking behavior, user-dependent PDR errors, and windowed FGO performance remains insufficiently explored.

Understanding this interaction is critical because PDR accuracy is inherently motion-dependent. Slow walking produces smaller acceleration variations and stable step patterns, whereas running introduces larger transient disturbances that increase PDR noise, step-length overestimation, and heading fluctuations. These differences indicate that one fixed window size cannot work well for all motions. A window that is suitable for slow or normal walking may be too large for fast motions like jogging or running, while a smaller window may not provide enough information for slower motions. Therefore, using a single window size for all conditions can either reduce accuracy or waste computation, depending on the type of motion.

Additionally, although previous studies have shown that FGO can reduce PDR errors, the resilience of FGO to motion-induced PDR outliers across different users has not been systematically examined. Variations in height, stride length, gait style, and walking speed may influence PDR uncertainty, and it remains unclear whether FGO consistently mitigates such errors more effectively than KF for a diverse group of users.

To address these gaps, this paper analyzes the relationship between pedestrian motion dynamics and the performance of FGO-based fusion. Specifically, we investigate:

- whether walking speed and gait variability influence the optimal sliding-window size in FGO.
- whether FGO consistently reduces PDR-induced outliers more effectively than KF across multiple users and motion types.

By addressing these questions, this work provides the first systematic investigation of how motion dynamics and user-specific gait characteristics jointly influence FGO-based fusion performance and window-size selection. The main contributions of this study are summarized as follows.

- Motion-dependent windowing: We empirically demonstrate that the optimal sliding-window size in FGO is motion-dependent, with slower motions benefiting from longer windows and dynamic motions achieving similar accuracy with shorter windows.
- Outlier suppression across users: We analyze the robustness of FGO to motion-induced PDR errors across ten pedestrians and show that FGO suppresses PDR outliers more effectively than KF under diverse gait behaviors.

The remaining part of the paper is structured as follows: Section 2 presents the methodology. Section 3 describes the experimental evaluation and setup. Section 4 details the analysis and results. Finally, Section 5 concludes the paper, summarizing the findings and future work.

## 2. Methodology

This section summarizes the fusion framework used in this study. Since the full mathematical formulation of the PDR algorithm, the KF-based PDR+GNSS fusion architecture, and the batch FGO-based PDR+GNSS architecture have already been presented in our earlier works [8,9,10], we avoid repeating those details here. Instead, this section provides a quick overview of FGO-based PDR+GNSS and a detailed description of the window-based FGO developed to analyse the walking behaviour of different pedestrians with FGO window sizes.

### 2.1. FGO-Based PDR+GNSS Fusion Architecture in Smartphone

A factor graph is a probability model that utilizes a graph to represent the probability dependence of different variables. Specifically, a factor graph is a bipartite graph composed of two types of nodes: the unknown state variables and the factor nodes, which represent the conditional probabilities of some set of state variables. The undirected edges connect the state variable nodes and corresponding factor nodes. There is an edge between the two nodes only when the variable node and the factor node have a functional relationship. A factor graph allows to decompose a global probability function into the product of multiple local probability functions, reducing the complexity of solving the global function.

In sensor integration, we are usually interested in estimating a set of state variables *X* from a set of given measurements *Z*. Assuming a Markov model, that conditional probability density function can be formulated:

(1) $$P\left(X|Z\right)=\prod_{i=1}^{k}\frac{P\left(z_{i}|x_{i}\right)P\left(x_{i}|x_{i-1},u_{i}\right)}{P(z_{i})}P\left(x_{0}\right)$$

where $z_{i}$ represents the measurements observed at epoch *i* (e.g., GNSS measurements), $x_{i}$ represents the system state at *i*, and $u_{i}$ denotes the control input (e.g., PDR measurements).

The most often used estimator for these unknown state variables *X* is the maximum a posteriori, or MAP estimate:

(2) $$\hat{X}=\arg\max P\left(X|Z\right)=\arg\max\prod_{i=1}^{k}P\left(z_{i}|x_{i}\right)P\left(x_{i}|x_{i-1},u_{i}\right)$$

In FGO-based integration, all these likelihood and transition probabilities are treated as the factorization of the global probability:

(3) $$\hat{X}=\arg\max\prod_{j=1}^{n}f\left(x_{j}\right)$$

Each factor models a constraint and must include a measure of uncertainty. The most common model is a Gaussian noise:

(4) $$P\left(z_{i}|x_{i}\right)\propto \exp\left(-\frac{1}{2}{∥h_{i}\left(x_{i}-z_{i}\right)∥}^{2}{▿}_{i}\right)$$

(5) $$P\left(x_{i}|x_{i-1}\right)\propto \exp\;\left(-\frac{1}{2}{∥\Phi_{i}\left(x_{i-1}\right)-x_{i}∥}_{\Omega_{i}}^{2}\right)$$

where the function $\Phi_{i}\left(\cdot \right)$ describes the relationship between the preceding states $x_{i-1}$ and $x_{i}$, the function $h_{i}\left(\cdot \right)$ represents the relationship between the state $x_{i}$ and the measurement $z_{i}$, and the covariance matrices are denoted by ${▿}_{i}$ and $\Omega_{i}$.

Taking the negative log and dropping the factor 1/2 transforms the problem into the minimization of an error function, that is, a nonlinear least-squares problem:

(6) $$\hat{X}=\arg\min\left(\sum_{i=1}^{k}{∥\Phi_{i}\left(x_{i-1}\right)-x_{i}∥}_{\Omega_{i}}^{2}+\sum_{i=1}^{k}{∥h_{i}\left(x_{i}\right)-z_{i}∥}_{{▿}_{i}}^{2}\right)$$

The major difference in comparison to KF is that FGO includes all historical observation measurements. Recent studies demonstrate that FGO outperforms traditional KF, and researchers are actively exploring the benefits of FGO in different navigation systems [3,5,7,11,12].

**Figure 1:**
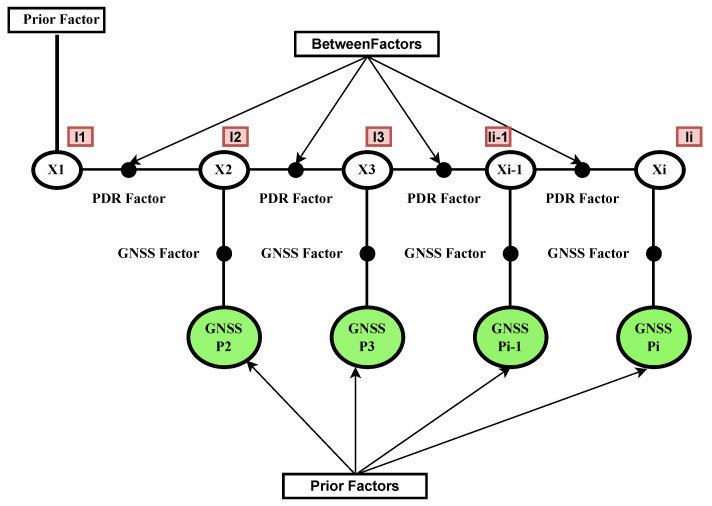
Illustration of factor graph construction for PDR+GNSS fusion in the FGO framework. Each node $X_{i}$ represents a pedestrian pose, connected via PDR-based between factors. GNSS observations are incorporated as unary factors associated with individual poses. A prior factor is set on the initial pose, and GNSS priors are included for graph consistency.

In our case, as shown in Figure 1, the factors represent the GNSS measurements and the transition between states due to the motion model and PDR measurements:

PDR Factor: This factor signifies the constraint enforced by the motion model derived from the PDR system.

(7) $$\begin{array}{c} {∥e_{k+1}^{PDR}∥}_{\sum_{k+1}^{PDR}}^{2}={∥X_{k+1}-f\left(X_{k}\right)∥}_{\sum_{k+1}^{PDR}}^{2}\\ ={∥x_{k+1}-\left(x_{k}+\left[\begin{array}{c} {\mathrm{SL}}_{t,t+1}\cdot \cos\left(\phi \right)\\ {\mathrm{SL}}_{t,t+1}\cdot \sin\left(\phi \right) \end{array}\right]\right)∥}_{\sum_{k+1}^{PDR}}^{2} \end{array}$$

It is important to note that variations in pedestrian motion (e.g., slow walking, normal walking, jogging, and running) are implicitly captured through the PDR measurements used in this factor. The step length ${\mathrm{SL}}_{t,t+1}$ and heading ϕ are directly estimated from smartphone IMU data and therefore naturally reflect motion-dependent gait characteristics as well as inter-user variability. No explicit motion-specific PDR model is employed, as the objective of this work is to analyze how motion-induced variations in PDR quality influence the performance of FGO and sliding-window configurations.

GNSS Factor: Similarly, this factor represents the constraint imposed by the GNSS measurements

(8) $${∥e_{k+1}^{GNSS}∥}_{\sum_{k+1}^{GNSS}}^{2}={∥X_{k+1}-GNSS_{k+1}∥}_{\sum_{k+1}^{GNSS}}^{2}$$

Therefore:

(9) $$\hat{X}=\arg\min\left(\sum_{i=1}^{k}\left[{∥e_{i+1}^{PDR}∥}_{\sum_{i+1}^{PDR}}^{2}+{∥e_{i+1}^{GNSS}∥}_{\sum_{i+1}^{GNSS}}^{2}\right]\right)$$

### 2.2. Sliding Window-Based FGO PDR+GNSS Fusion Architecture

In batch FGO, the optimizer reprocesses all historical measurements at each update, causing the factor graph and computation time to grow with trajectory length. While this full-batch strategy yields high accuracy, it becomes computationally expensive on smartphones, motivating the use of a sliding-window approach that limits optimization to a fixed number of recent states. As the trajectory grows, the Levenberg–Marquardt solver must operate on an increasingly large state vector and Jacobian matrix at each GNSS update, leading to a steady increase in computational cost. Therefore, a sliding-window optimization strategy is needed to restrict the number of factors considered during each iteration and reduce processing time.

The sliding-window approach addresses this by maintaining a fixed-size window of recent poses. At each GNSS measurement, the factor graph is reconstructed using only the most recent *N* poses and their associated factors, while information outside the window is discarded. This ensures computational feasibility but sacrifices long-term consistency because older states are no longer optimized once they fall outside the window. In other words, no marginalization is performed in this approach.

We intentionally adopt this naïve fixed-lag smoother without marginalization for two reasons. First, our goal is to isolate the influence of window length on FGO performance without introducing additional approximations from the marginalization process. Second, prior work has shown that marginalization introduces cross-correlations that depend on implementation choices and may complicate the analysis of motion-dependent behaviour. For this reason, we discard factors outside the window directly and assign a simple prior to the oldest retained state, ensuring a clean and interpretable evaluation of window-size effects.

Problem Formulation: The optimization problem for the sliding window can be formulated as:

(10) $${\hat{X}}_{k-N:k}=\arg\max P\left(X_{k-N:k}|Z_{k-N:k}\right),$$

where:

- ${\hat{X}}_{k-N:k}$ is the estimated trajectory for the most recent *N* poses within the sliding window,
- $Z_{k-N:k}$ represents the set of measurements (e.g., GNSS and PDR) available in the window.

Using Bayes’ rule and assuming a Markovian structure for the motion dynamics, the problem simplifies to:

(11) $${\hat{X}}_{k-N:k}=\arg\max\prod_{i=k-N}^{k}P\left(z_{i}|x_{i}\right)P\left(x_{i}|x_{i-1},u_{i}\right),$$

where:

- $P(z_{i}|x_{i})$: Likelihood of the measurement $z_{i}$ (e.g., GNSS) given the state $x_{i}$,
- $P(x_{i}|x_{i-1},u_{i})$: Transition probability between consecutive poses $x_{i-1}$ and $x_{i}$, based on odometry $u_{i}$.

Factor Graph Construction: At each inclusion of a new GNSS factor, the sliding-window optimization involves constructing a factor graph containing:

1. Prior Factor: A prior factor is applied to anchor the oldest pose in the window ($x_{k-N}$) to maintain observability:
  (12) $$P\left(z_{i}|x_{i}\right)∼\exp\left(-\frac{1}{2}{\left(x_{i}-z_{i}\right)}^{⊤}\Sigma^{-1}\left(x_{i}-z_{i}\right)\right),$$
  where $z_{i}$ is the GNSS measurement and Σ is its noise covariance.
2. GNSS Factors: GNSS measurements are treated as priors for all poses within the window to ensure alignment with global positioning.
3. PDR Position Factors: The relative motion between consecutive poses is modeled as:
  (13) $$P\left(x_{i}|x_{i-1}\right)\propto \exp\left(-\frac{1}{2}{∥\Phi_{i}\left(x_{i-1}\right)-x_{i}∥}_{\Omega_{i}}^{2}\right),$$
  where $\Phi_{i}\left(x_{i-1}\right)$ represents the predicted transformation between $x_{i-1}$ and $x_{i}$ based on PDR, and $\Omega_{i}$ is the corresponding information matrix.

Optimization Process: At each iteration, a new factor graph is constructed using the *N* most recent poses and their associated factors (GNSS and PDR). In both FGO and sliding windowing, the optimization process follows a similar structure. The constructed factor graph is optimized using the LM algorithm, which iteratively refines the state estimates to obtain the MAP solution. In sliding windowing, only the most recent *N* poses are considered, ensuring a computationally efficient update while still preserving the key constraints. After optimization, the estimated trajectory is updated, with only the most recent pose retained for further evaluation.

**Figure 2:**
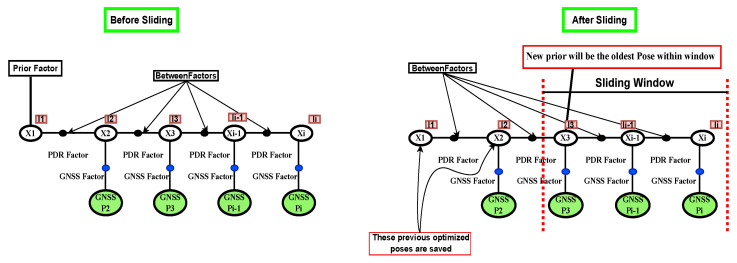
Sliding window FGO mechanism: Only the most recent poses and factors are retained for optimization, while older poses are fixed and a new prior is assigned to the oldest pose within the window.

Figure 2 illustrates the sliding-window mechanism used in our implementation. Only the most recent poses and their associated GNSS and PDR factors are retained in the optimization window. When a state moves outside the window, both the state and its connected factors are removed, and a simple prior is assigned to the oldest state that remains within the window. Unlike marginalization-based fixed-lag smoothers, no Schur-complement marginalization is performed. This naïve windowing strategy allows us to isolate the effect of window length on FGO performance without introducing additional approximations or correlations from marginalization.

#### Implementation and Optimization

This research utilizes the Georgia Tech Smoothing and Mapping (GTSAM) library [13] for implementing the FGO-based PDR+GNSS fusion framework. GTSAM is a widely used C++ library designed for solving large-scale factor graph-based optimization problems, particularly in SLAM and sensor fusion domains. Its modular design and efficient solvers make it well-suited for real-time state estimation tasks.

In our implementation, GTSAM is used to construct and solve factor graphs representing the fusion of PDR and GNSS data. Each node in the graph corresponds to a pedestrian pose, and factors are added based on GNSS position updates, PDR constraints (e.g., step length and heading), and odometry priors. The LM optimizer is employed to solve the nonlinear least-squares problem at each optimization step. To support sliding-window optimization, the factor graph is rebuilt at each GNSS epoch, incorporating only the latest *N* poses and their associated measurements. This fixed smoothing approach allows efficient state estimation while controlling computational complexity. No marginalization is applied in the windowing approach; older states are simply discarded when they fall outside the window. All experiments, including both batch and windowed FGO variants, were implemented and executed using GTSAM’s flexible graph-building tools and solver configuration utilities.

## 3. Experimental Description

To conduct the experiments, we employed a Huawei Y8P smartphone to collect IMU data and obtain positioning information from its onboard GNSS receiver. The device integrates a chip-scale GNSS module capable of tracking multiple constellations, with detailed specifications provided in the manufacturer’s datasheet [14].

For ground truth reference, we utilized a high-precision Ublox ANN-MB-00-00 L1/L2 multi-band GNSS antenna paired with the C099-F9P application board, which incorporates the ZED-F9P-01B-00 GNSS module. This configuration enabled centimeter-level positioning accuracy by leveraging multi-frequency, multi-constellation data from GPS, Galileo, GLONASS, and BeiDou systems.

**Figure 3:**
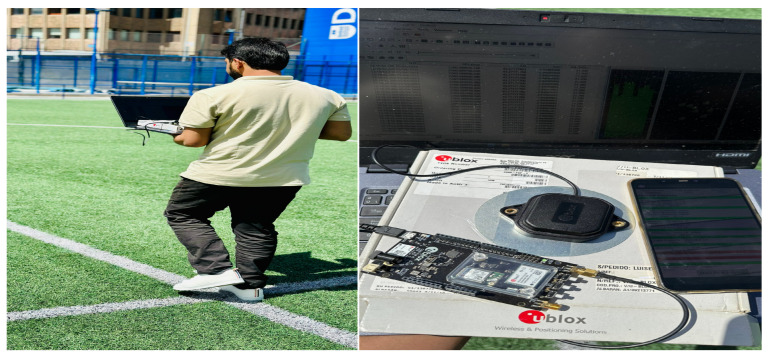
Data collection and experimental setup: (**left**) pedestrian carrying the smartphone during outdoor data collection; (**right**) ground-truth GNSS setup consisting of the Ublox ANN-MB-00-00 antenna connected to the ZED-F9P processing board.

Data collection was carried out in an outdoor environment on a football field located at the University of Deusto, Bilbao, Spain. A sharp rectangular trajectory of approximately 100 m was chosen for testing, bounded by tall buildings that partially obstructed satellite visibility. This urban setting introduced GNSS noise, providing a realistic and challenging scenario for evaluating fusion performance. A visual overview of the data collection environment and device setup is presented in Figure 3.

### Data Collection and Processing

Smartphone IMU and GNSS Data: The GetsensorsData app [15] was used to record IMU readings and GNSS position coordinates from the smartphone. During the experiments, each participant held the device in portrait orientation, aligned with their direction of movement to ensure consistent data capture. A total of 40 datasets were collected from 10 participants with diverse heights and genders, performing four distinct motion types: slow walking, normal walking, jogging, and running, as detailed in Table 1.

Each participant completed two full laps along a predefined rectangular path. While individuals were free to select their natural walking or running pace, all were instructed to maintain the phone in portrait mode, positioned centrally at chest level throughout the trials. Before data collection, participants received a demonstration to standardize execution across different motion categories. To classify each data into slow walking, normal walking, jogging, or running, predefined upper speed limits were used during offline processing, as listed in Table 2. These thresholds were employed only for motion-type classification, while participants moved freely at their natural pace.

Ground Truth Data: High-precision ground truth data were acquired using a Ublox ANN-MB-00-00 L1/L2 multi-band GNSS antenna, capable of receiving signals from GPS, GLONASS, Galileo, and BeiDou constellations. The antenna was connected to a ZED F9P-01B-00 processing board and interfaced via Ublox’s U-center 22.08 software for data logging and management. The reference trajectory was recorded separately for each motion session only to ensure precise temporal alignment and consistent GNSS conditions during each experimental run. This does not impose any constraint on the participant’s speed; pedestrians always moved at their natural “own will” pace. The ground truth collection simply provides a high-accuracy geometric reference for each experimental pass over the same path. The integration of multi-constellation and dual-frequency capabilities enabled a horizontal positioning accuracy exceeding 2.5 cm, providing a reliable reference framework for performance evaluation and analysis.

The ZED-F9P receiver provides high-precision GNSS positioning using dual-frequency, multi-constellation observations and its built-in positioning engine. In this study, the ground truth positions were obtained directly from the receiver’s GNSS solution outputs (latitude and longitude) logged via the u-center software, without implementing additional post-processing or custom PPP/RTK algorithms. Under open-sky conditions, this configuration provides centimetre-level horizontal accuracy (on the order of a few centimetres), which is sufficient to serve as a reliable ground truth reference for evaluating the proposed fusion methods.

Timely Synchronization of Ground Truth and Smartphone data: Since we collected our ground truth data separately for each type of movement, we needed to make sure the time records of this data matched up with the GNSS data we gathered from the 10 users’ smartphones. To make this happen, we followed a set of detailed steps:

- Consistent Time Basis: Both the ground-truth GNSS data and the smartphone PDR+GNSS data were recorded using GNSS timestamps, providing a common temporal reference.
- Timestamp Alignment and Interpolation: Since the ground-truth GNSS receiver and the smartphone sensors operated at different sampling rates, offline interpolation was applied to align all measurements to a common time base. This ensured that ground-truth positions, smartphone GNSS updates, and PDR estimates corresponded to the same time instants for evaluation.
- Device Co-location: During data collection, the Ublox ground-truth GNSS antenna and the smartphone were placed immediately adjacent to each other. This close physical placement ensured nearly identical GNSS signal conditions.

Finally, we validated the synchronization process by conducting a thorough cross-check. Ensure that the GNSS data from the smartphones aligns seamlessly with the ground truth dataset, with both datasets consistently referencing the same moments at the seconds level.

Fusion Methods Parametrization The efficacy of the FGO and KF fusion algorithms hinges on their adept utilization of parameter covariance settings. Throughout this study, we preserved uniform parameter settings for all pedestrian motions across the comprehensive dataset comprising forty instances. In the case of the KF, its parametrization in our case, we set the KF parameters as follows:

$$P=\left[\begin{array}{cc} 0 & 0\\ 0 & 0 \end{array}\right],\;Q_{k}=\left[\begin{array}{cc} 1 & 0\\ 0 & 1 \end{array}\right],\;R=\left[\begin{array}{cc} 2 & 0\\ 0 & 2 \end{array}\right]$$

where *P* is the initial state covariance matrix, $Q_{k}$ is the process noise covariance matrix, and *R* is the measurement noise covariance matrix. We selected these values for specific reasons. They signify that our initial estimate is highly reliable and characterized by a significant level of certainty. The parameters for the process noise covariance matrix indicate that the system noise is anticipated to be both minimal and uncorrelated. Moreover, the measurement noise covariance matrix, with non-zero values in the off-diagonal positions and zeros in the diagonal, is a clear indicator of noise present in the sensor measurements.

In the domain of FGO, our approach involves two main factors i.e., PDR and GNSS. It is important to recognize that both of these factors come with uncertainties and noise. To handle these uncertainties, we have used GTSAM’s odometry and prior noise model classes. For PDR, which deals with measurements affected by different motions and users, we have set bit higher values for horizontal positions (X and Y). This decision acknowledges the higher uncertainties associated with PDR measurements in our context. Conversely, GNSS measurements are more stable in our scenario, so we have used lower values. This is because we understand that GNSS measurements have lower uncertainties compared to PDR in our specific case. This precision in our approach enables us to effectively manage and mitigate uncertainties and noise, ultimately enhancing the accuracy and robustness of our analytical processes.

odometryNoise=noiseModel.Diagonal.Sigmas([2;2;0.1])

priorNoise=noiseModel.Diagonal.Sigmas([0.1;0.1;0.1])

## 4. Results and Discussion

This section presents the experimental results and provides an integrated interpretation of how pedestrian motion dynamics, inter-user variability, and sliding-window configuration influence the performance of the PDR+GNSS fusion architecture. In particular, the analysis examines how variations in gait behaviour affect the quality of the PDR block and, consequently, the relative performance of KF and FGO, and then explores how different window lengths in the sliding-window FGO framework balance temporal modelling capability with computational efficiency. To this end, the results are organised into two parts. The first compares KF and FGO across multiple motions and pedestrians, and the second evaluates the impact of window size on Sliding-Window Factor Graph Optimization (SWFGO) performance under the same motion conditions.

### 4.1. Comparison of FGO and KF Across Different Pedestrian Motions and Users

**Figure 4:**
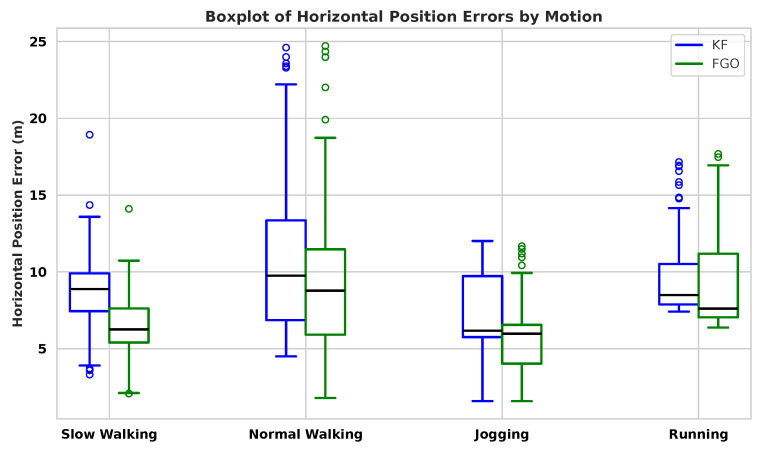
Boxplot comparison of HPEs between KF and FGO across four pedestrian motions. FGO exhibits narrower interquartile ranges and fewer outliers, indicating reduced estimator variance and improved localization stability.

Figure 4 shows the Horizontal Position Error (HPE) distributions for KF and FGO across slow walking, normal walking, jogging, and running. FGO achieves narrower interquartile ranges and fewer extreme values in all motions, indicating a clear reduction in error variability. This behaviour can be explained by the characteristics of the PDR input. Slow walking and jogging usually produce more regular step patterns and smoother heading changes, leading to smaller PDR disturbances that FGO can smooth more effectively. As a batch optimizer, FGO updates the entire trajectory when new measurements arrive, allowing inconsistencies to be spread over multiple time steps. In contrast, KF updates the state recursively and only uses the most recent measurement. When PDR noise suddenly increases due to mid-stride heading jitter or step-length overestimation the KF output shows larger instantaneous errors because it cannot adjust or correct the past states once they have been estimated.

**Figure 5:**
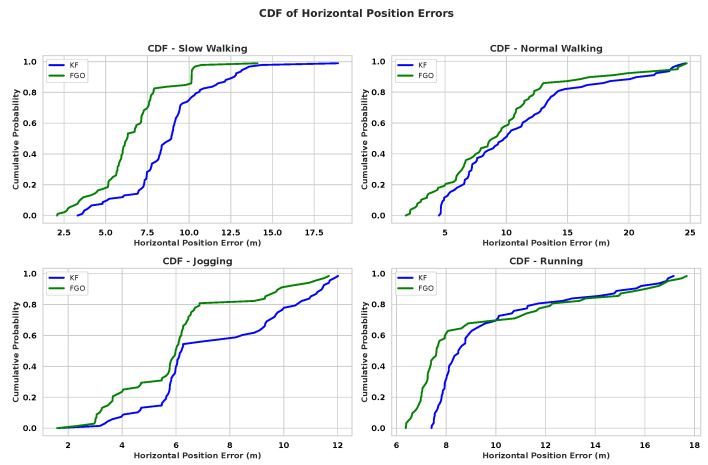
CDF of HPEs for KF and FGO across four pedestrian motions. FGO curves show a consistent leftward shift, indicating a higher proportion of low-error estimates compared with KF.

The CDF curves in Figure 5 further support these observations. In all four motions, the FGO curve is consistently shifted to the left of the KF curve, meaning that a larger proportion of FGO estimates fall within lower error ranges. The improvement is most evident in slow walking and jogging, where FGO achieves substantially lower errors at the 90th-percentile level. Running remains more challenging, although FGO still improves the mean and median errors, the high-frequency gait dynamics and stronger inertial disturbances reduce its ability to suppress large PDR deviations. Under these conditions, even small heading fluctuations or step-length errors propagate more strongly through the fused trajectory, limiting the smoothing advantage of FGO.

Table 3 summarises the statistical measures. The most significant gains appear in slow walking (mean improvement 25.29%) and jogging (19.02%), where PDR noise is structured and more predictable. Normal walking shows moderate improvements (13.73%). Running exhibits limited improvement in standard deviation, indicating that the more irregular PDR behaviour at higher speeds restricts the smoothing advantage of FGO.

These results highlight two important observations. First, the performance of the fusion is not determined by the fusion method alone, but it is strongly modulated by the quality of the PDR input, which varies with gait dynamics. Second, FGO is particularly effective when PDR noise is temporally correlated or evolves smoothly, conditions under which global smoothing reconstructs more consistent trajectories than a recursive filter.

**Figure 6:**
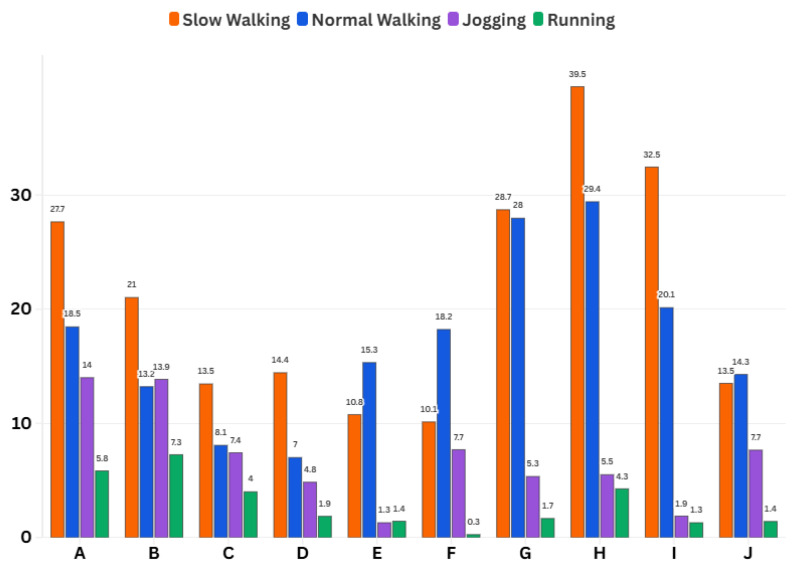
Mean 2D HPE reduction achieved by FGO relative to KF for different motion types and pedestrians.

**Figure 7:**
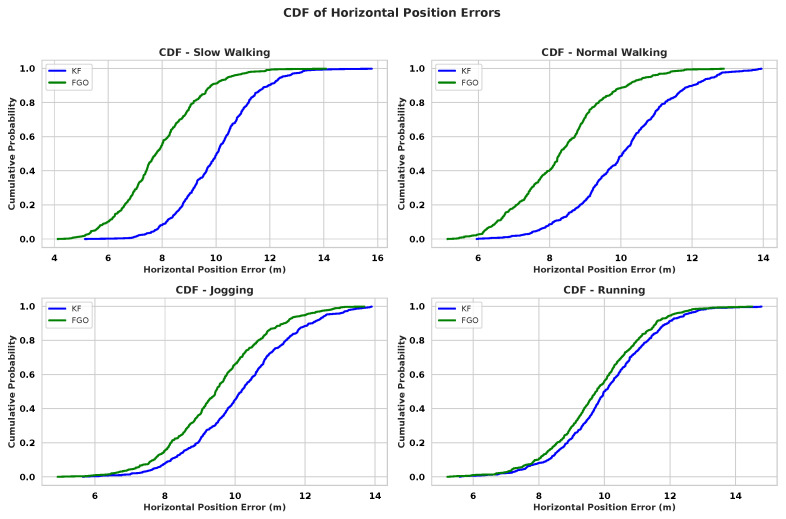
CDF of horizontal position errors (HPEs) aggregated over all pedestrians for different motion types: slow walking; normal walking; jogging; running. FGO consistently achieves higher cumulative probabilities at low error thresholds compared with KF, despite inter-subject variability.

**Figure 8:**
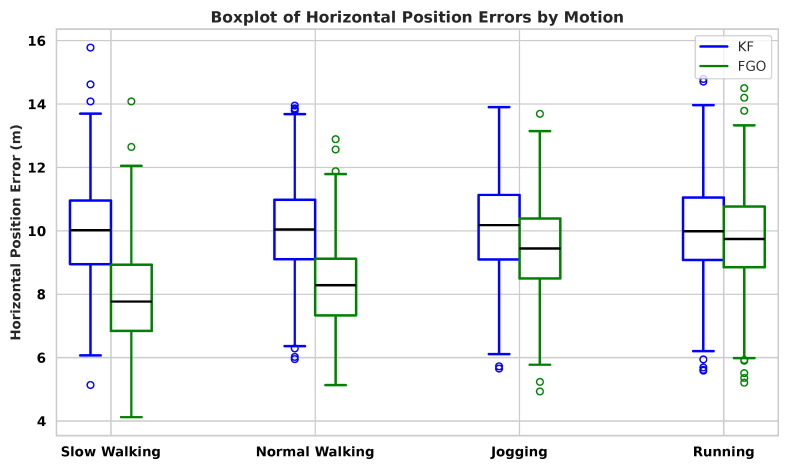
Boxplot of HPEs over ten pedestrians and four motions. FGO shows lower medians and reduced spread across all subjects, indicating improved robustness to inter-subject variability.

The inter-user analysis (Figure 6, Figure 7 and Figure 8) further supports this dependency. Although FGO consistently outperforms KF for all pedestrians, the amount of improvement varies noticeably between individuals. These differences arise because each user has a distinct walking pattern, including variations in step length, step frequency, upper-body movement, and how the smartphone is carried or held. Users with smoother and more regular gait patterns tend to show larger benefits from FGO, whereas users with irregular steps or more pronounced body motion experience smaller improvements. Even with this diversity, the aggregated boxplots across all subjects show clearly lower medians and reduced spreads for FGO, indicating that it provides more stable performance across heterogeneous pedestrians. Overall, these results show that FGO mitigates many user-dependent and motion-dependent PDR disturbances better than KF.

### 4.2. Comparison of FGO and SWFGO Across Different Window Sizes, Pedestrians, and Motions

The previous subsection demonstrated that FGO improves over KF but requires substantial computational cost due to its batch nature. We now examine how sliding-window FGO (SWFGO) behaves for different window sizes and how it balances temporal modelling capability with computational feasibility.

#### 4.2.1. Effect of Window Size on PDR+GNSS Fusion

**Figure 9:**
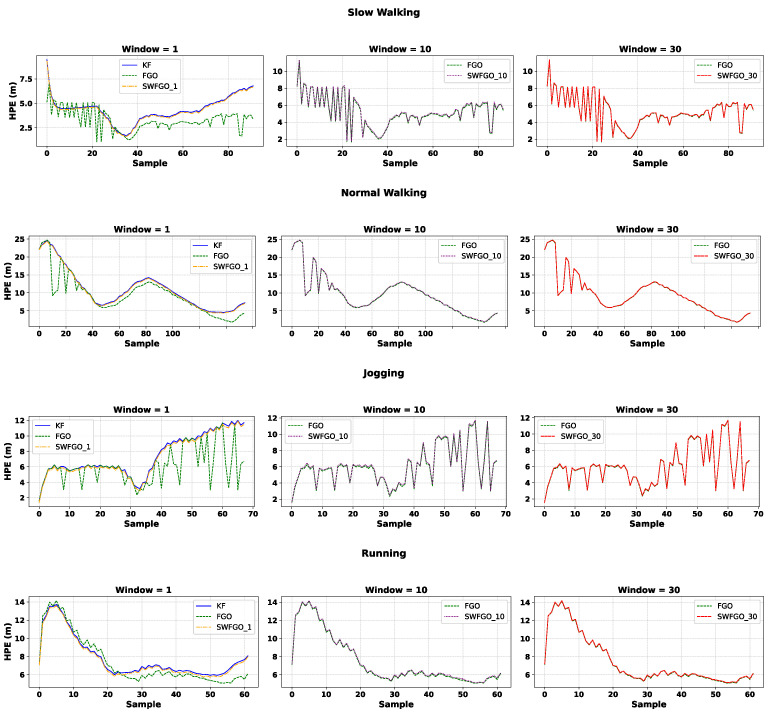
HPE time series across four motion types: slow walking, normal walking, jogging, and running. Each subfigure compares KF, batch FGO, and SWFGO with window sizes of 1, 10, and 30.

Figure 9 presents the HPE time series for KF, FGO, and SWFGO with window sizes of 1 pose, 10 poses, and 30 poses for the four motion types. The case w=1 pose is of particular interest because it mimics a recursive estimator. It uses only the most recent pose in the optimization window. Although this configuration still benefits from graph modelling, it captures insufficient temporal structure to match the behaviour of larger windows. As a result, SWFGO (*w* = 1 pose) shows only slight improvement over KF.

For w=10 poses and w=30 poses, SWFGO retains enough temporal information to exploit the correlations in PDR and GNSS measurements. In slow and normal walking, where PDR errors evolve gradually, larger windows provide clear benefits: the trajectories become smoother and more consistent, closely approaching batch FGO. In jogging and running, the benefit of increasing the window size is still present but less pronounced, as the higher-frequency gait dynamics reduce the effective correlation time in the motion model. Under these conditions, shorter windows already capture most of the useful temporal structure. From a physical standpoint, the PDR increments are affected by gait-dependent disturbances. During slow and normal walking, step timing and phone orientation changes are typically smoother, so step-length and heading errors evolve more gradually and remain temporally correlated over multiple steps. In this case, a larger sliding window allows SWFGO to exploit cross-step consistency by jointly optimizing several consecutive poses, effectively distributing PDR errors over time and producing smoother trajectories. In jogging and running, higher-frequency body motion and stronger arm and torso dynamics introduce larger short-term heading jitter, step-length variability, and occasional irregular steps, reducing the effective correlation length of the PDR errors. As a result, increasing the window beyond a moderate size yields diminishing returns, because additional historical states contribute less coherent information for smoothing. While the sensitivity to window length differs across motions, our results indicate that an intermediate window (around 10 poses) provides a consistently favorable accuracy-efficiency balance across all tested motion types.

Table 4 quantifies these trends. Larger window sizes (w=10 poses and w=30 poses consistently reduce the mean error compared with KF and with the minimal configuration w=1 pose. Their performance approaches that of batch FGO across all motions, although they do not surpass the batch solution. This indicates that using a limited temporal context (10-30 poses) can achieve accuracy close to full-graph optimisation, while avoiding the high computational cost associated with batch FGO.

#### 4.2.2. Trade-Off Between Accuracy and Computational Load

**Figure 10:**
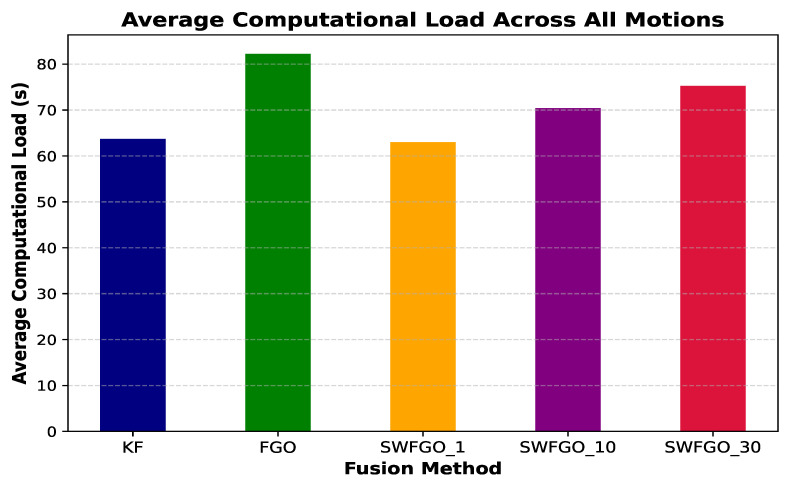
Average total computational time across all motion types for KF, SWFGO (1 pose), SWFGO (10 poses), SWFGO (30 poses), and batch FGO. KF and SWFGO with a 1-pose window require the lowest computational load, while batch FGO incurs the highest. Intermediate window sizes (10 and 30 poses) show predictable and bounded computational demands, reflecting the increased number of states included in the optimization.

To evaluate the balance between positioning accuracy and computational effort, we analysed the total computational time required to process each trajectory for all fusion methods. Since each method processes the same number of GNSS measurements per motion, the total runtime provides a consistent and comparable measure of computational cost. Figure 10 presents the average computation time across all motion types. KF and SWFGO (w=1) pose are the most efficient, requiring the lowest overall processing time. Increasing the window size to w=10 poses and w=30 poses introduces higher but still bounded computational load because more states and factors must be optimised at each GNSS update. Batch FGO incurs the highest computational cost because the full factor graph is re-optimised at every GNSS update.

**Figure 11:**
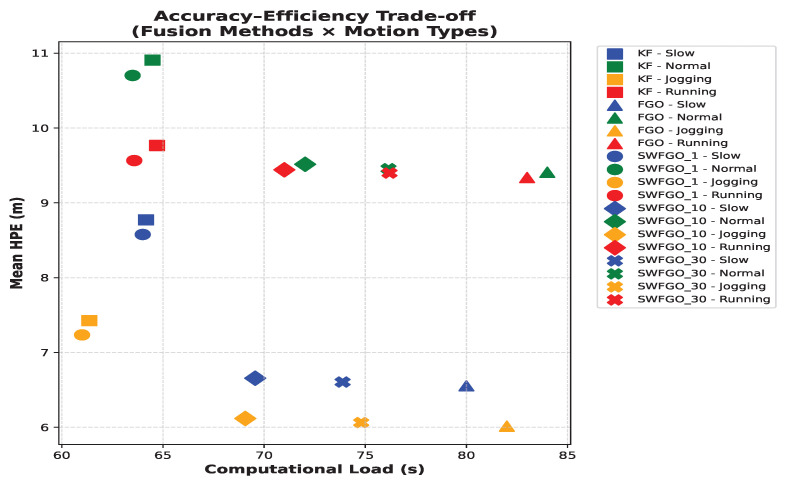
Accuracy–efficiency trade-off using average mean HPE and average computational load across all motion types. Each point represents one fusion method. KF resides in the low-cost/high-error region, whereas batch FGO achieves the lowest error at the highest cost. SWFGO with w=10 poses and w=30 pòses achieves accuracy close to batch FGO while requiring substantially less computation, demonstrating a favourable compromise for real-time smartphone-based pedestrian navigation.

While Figure 10 shows the aggregated computational requirements, it is equally important to understand how this relates to positioning accuracy. The computational load of SWFGO increases with window size because the number of optimized states and associated factors grows linearly with the window length. Increasing the window from 10 to 30 poses approximately triples the number of poses included in each optimization, resulting in larger Jacobian matrices and increased solver iterations. Although this leads to a noticeable increase in computation time, the corresponding reduction in mean HPE is marginal (Table 4). This quantitative behavior explains why SWFGO with a 10-pose window achieves a more favorable accuracy-efficiency trade-off than larger windows. Figure 11 summarises the accuracy-efficiency trade-off by plotting the average mean HPE against the average computational load for each fusion method. KF occupies the low-cost/high-error region, whereas batch FGO lies in the high-cost/low-error corner. SWFGO (w=1) poses provides only modest accuracy improvement over KF at a similar computational cost. In contrast, SWFGO (w=10) poses and SWFGO (w=30) poses achieve accuracy close to batch FGO while requiring substantially less computation. This behaviour is consistent across all motion types and indicates that intermediate window sizes serve as an effective compromise between accuracy and efficiency in practical pedestrian navigation systems.

### 4.3. Summary of Findings

Overall, the results confirm that the behaviour of the PDR block, shaped by both user gait and motion dynamics, strongly influences the fusion performance. FGO and SWFGO mitigate many PDR-induced disturbances that KF cannot correct. The sliding-window analysis shows that increasing the window from 1 to 10 poses provides the largest accuracy improvement, while further increasing it to 30 poses yields only marginal gains. This pattern is consistent across slow, normal, jogging, and running motions, indicating that an intermediate window of approximately 10 poses offers a stable accuracy–efficiency compromise rather than a motion-dependent optimum.

The experimental findings highlight three main insights. First, pedestrian motion dynamics strongly modulate PDR quality and therefore affect how KF and FGO perform. Motions with smoother and more regular gait patterns provide conditions where FGO’s smoothing capability yields the greatest improvements. Second, inter-user variability introduces additional PDR disturbances that KF cannot effectively handle. At the same time, FGO consistently reduces both the frequency and magnitude of such errors across users with different gait behaviours. Third, the temporal context captured by the sliding window plays a critical role: increasing the window size from 1 to 10 poses leads to clear improvements across all motion types, while further increasing to 30 poses produces only marginal gains. From an estimation perspective, this behavior can be explained by the different ways in which KF and FGO propagate uncertainty over time. The KF follows a recursive update scheme in which the current state estimate is primarily influenced by the previous state and the most recent measurements, with past information summarized through a propagated covariance. In contrast, FGO performs smoothing by jointly optimizing multiple states within the sliding window, allowing measurement information from several epochs to directly constrain the current estimate. Increasing the window size therefore increases the number of states and factors participating in the optimization, strengthening the influence of historical information and reducing sensitivity to local PDR disturbances. However, as the window grows, additional historical states contribute progressively less new independent information, while the computational cost continues to increase. This explains why expanding the window from 1 to 10 poses yields substantial accuracy gains, whereas further increasing it to 30 poses results in diminishing returns. The trends do not indicate a motion-dependent optimal window size; instead, an intermediate window (10 poses) provides a stable balance between accuracy and computational cost for slow, normal, jogging, and running motions alike. Together, these insights support practical, computationally efficient windowed-FGO configurations for real-time smartphone pedestrian navigation.

## 5. Conclusions and Future Work

This study investigated the behaviour of FGO-based PDR+GNSS fusion under diverse pedestrian motions and inter-user variability, and examined how sliding-window configurations influence the trade-off between localization accuracy and computational efficiency. Unlike conventional analyses that focus solely on average performance, this work provided a deeper exploration of motion-dependent and user-dependent factors that shape PDR quality and, consequently, the performance of KF, FGO, and SWFGO.

Across all motion types and pedestrians, FGO consistently achieved lower mean and median horizontal positioning errors than KF, while also reducing variance and suppressing large PDR-induced deviations. These improvements were most pronounced in slow walking and jogging, where PDR noise exhibits stable, temporally correlated behaviour that FGO can effectively smooth. Even in high-dynamic motions such as running, where inertial disturbances and stride variability challenge both PDR and GNSS FGO, maintained better central accuracy than KF, demonstrating its robustness in non-ideal conditions.

The sliding-window analysis revealed that the temporal context captured in the optimization window strongly influences performance. Larger windows (10–30 poses) provided accuracy close to batch FGO in slow and moderate motions, where long-term correlations are present, while smaller windows were sufficient in highly dynamic motions where the effective correlation time is shorter. The sliding-window analysis revealed that accuracy improves as the window size increases, with SWFGO using 10 poses offering a strong and consistent accuracy–efficiency balance across all motion types. Increasing the window to 30 poses produced only marginal additional accuracy gains while noticeably increasing computation. Importantly, the results do not indicate a motion-dependent optimal window size; instead, the same intermediate window configuration performed reliably for slow walking, normal walking, jogging, and running alike.

Overall, the results show that FGO and SWFGO not only outperform KF in accuracy but also demonstrate a superior ability to mitigate user and motion-dependent PDR errors. This work therefore provides a foundation for designing adaptive and computationally efficient pedestrian navigation systems that can operate reliably across diverse users and motion conditions. From a practical perspective, these findings suggest that a fixed intermediate sliding-window size (approximately 10 poses) offers a robust accuracy–efficiency compromise across a wide range of pedestrian motions, making it suitable for real-time smartphone-based navigation systems with limited computational resources. While different motion types exhibit varying sensitivity to window length, the consistent performance of this intermediate window suggests that reliable operation can be achieved without frequent reconfiguration. These findings also motivate adaptive sliding-window strategies that dynamically adjust the window length based on detected motion dynamics or estimated PDR uncertainty, which represents a promising direction for future real-time implementations.

Future work may explore the following directions:

- Developing adaptive sliding-window mechanisms that adjust window size in real time based on detected motion dynamics or PDR uncertainty.
- Integrating other sensors (e.g., barometric, camera, magnetometer) to improve robustness in urban or GNSS-challenged environments.
- Extending the analysis to indoor–outdoor transition scenarios and more diverse pedestrian populations to evaluate generalization.

## Figures and Tables

**Table 1:** Details of participants involved in the data collection, including height, gender, and pace.

Pedestrian	Height (cm)	Gender	Pace
A	155	Female	
B	160	Female	
C	157	Male	
D	150	Male	
E	147	Male	
F	140	Male	
G	180	Male	Own Will
H	185	Male	
I	188	Male	
J	168	Male	

**Table 2:** Defined upper pace limits for each pedestrian motion type used in the experiments. These thresholds were applied to differentiate between slow walking, normal walking, jogging, and running.

Motion Type	Pace Upper Limit
Slow Walking	1.5 m/s
Normal Walking	2.5 m/s
Jogging	4.5 m/s
Running	6.5 m/s

**Table 3:** Statistical summary of HPE performance metrics for KF and FGO under different pedestrian motions. Percent improvement is computed relative to KF.

Motion	Mean			Median			STD			90th Percentile		
	KF	FGO	%	KF	FGO	%	KF	FGO	%	KF	FGO	%
Slow Walking	8.77	6.55	25.29	8.88	6.26	29.57	2.64	2.23	15.52	12.46	10.15	18.56
Normal Walking	10.91	9.41	13.73	9.76	8.78	10.03	5.46	5.40	1.03	20.23	16.25	19.70
Jogging	7.43	6.01	19.02	6.17	5.98	3.09	2.64	2.34	11.47	11.30	9.73	13.93
Running	9.77	9.34	4.38	8.49	7.61	10.36	2.83	3.39	−19.71	14.84	15.56	−4.82

**Table 4:** Mean HPE (m) for KF, FGO, and SWFGO variants (window sizes 1, 10, and 30) across different motion types and the Combined row reports the mean HPE computed by aggregating all ten pedestrians across all four motion types.

Motion	KF	FGO	SWFGO (*w* = 1)	SWFGO (*w* = 10)	SWFGO (*w* = 30)
Slow Walking	8.77	6.56	8.58	6.66	6.61
Normal Walking	10.91	9.41	10.70	9.52	9.46
Jogging	7.43	6.02	7.24	6.12	6.06
Running	9.77	9.34	9.57	9.44	9.40
Combined	10.04	8.83	9.84	8.93	8.88

## Data Availability

The data that support the findings of this study are available from the corresponding author, upon reasonable request.

## Abbreviations

The following abbreviations are used in this manuscript:

FGO	Factor Graph Optimization
SWFGO	Sliding Window-Based FGO
LM	Levenberg–Marquardt
GNSS	Global Navigation Satellite Systems
GTSAM	Georgia Tech Smoothing and Mapping
IMU	Inertial Measurement Unit
KF	Kalman Filter
NLOS	Non-Line of Sight
PDR	Pedestrian Dead Reckoning
